# Superior Mesenteric Artery Injury during Robotic Radical Nephrectomy: Scenarios and Management Strategies

**DOI:** 10.3390/jcm12020427

**Published:** 2023-01-05

**Authors:** Aref S. Sayegh, Luis G. Medina, Anibal La Riva, Laura C. Perez, Jaime Poncel, Edward Forsyth, Giovanni E. Cacciamani, Ben Challacombe, Michael Stifelman, Inderbir Gill, Rene Sotelo

**Affiliations:** 1 The Catherine and Joseph Aresty Department of Urology, USC Institute of Urology, Keck School of Medicine, University of Southern California, Los Angeles, CA 90033, USA; 2 Department of Urology, Guy’s and St Thomas NHS Foundation Trust, London SE1 9RT, UK; 3 Department of Urology, Hackensack Meridian School of Medicine, Hackensack, NJ 07601, USA

**Keywords:** superior mesenteric artery injury, radical nephrectomy, intraoperative complications, robotics, management

## Abstract

Injury to the superior mesenteric artery (SMA) is a rare, underreported, and potentially devastating complication. This study aims to propose a systematic workup to describe how to prevent and manage SMA injury in a standardized stepwise manner. Three different instances of intraoperative injury to the SMA are described in an accompanying video. All three occurred when the SMA was misidentified as the left renal artery during left robotic radical nephrectomy. In the first case, the SMA was mistakenly identified as the renal artery, but after further dissection, the real renal artery was identified and SMA injury was prevented. In the second case, the SMA was clipped and the real left renal artery was subsequently identified, requiring clip removal. In the third case, the SMA was clipped and completely transected, requiring prompt repair by vascular surgery with a successful outcome. This study aims to propose a systematic workup to describe how to prevent and manage SMA injury in a standardized stepwise manner. The proper anatomic recognition of the SMA may prevent its injury. Intraoperative SMA injury should be promptly identified and repaired to avoid its devastating consequences.

## 1. Introduction

Robotic-assisted radical nephrectomy has become an increasingly popular option for the treatment of renal masses [1,2,3]. Robotic nephrectomy is more commonly performed via a transperitoneal approach due to anatomical familiarity and adequate working space [4]. The retroperitoneal approach represents a valid alternative, especially for posterior-laterally located renal masses, as it allows direct access to the renal artery without the need for colon mobilization [4].

There are several advantages of robotic technology to renal surgery, including an enhanced degree of freedom, three-dimensional visualization, higher magnification, surgeon’s ergonomics, the use of fluorescence imaging, and the elimination of tremors leading to a widespread utilization to perform more complex surgeries [5,6,7,8,9,10]. However, there is a potential risk for catastrophic complications during complex robotic renal surgery due to the close proximity of vital vascular structures [11,12,13,14,15].

Injury to the Superior Mesenteric Artery (SMA) is a rare, underreported, and potentially devastating complication. It can occur in patients with large left renal tumors, bulky lymphadenopathy, or in the setting of re-do surgery with significant retroperitoneal and intraabdominal scarring, which may distort the vascular anatomy. In most cases, inadvertent injury occurs due to misidentifying the SMA as the left renal artery (LRA) [16,17].

Failure to recognize and promptly repair an SMA injury may result in ischemic bowel and mortality in up to 50% of the cases [11,18]. Therefore, it is imperative that surgeons are able to recognize and manage this potentially life-threatening complication.

This study describes three different scenarios of injury to the SMA when misidentified as the LRA during left robotic radical nephrectomy with an accompanying video. A systematic workup is presented to describe how to prevent and manage SMA injury in a standardized stepwise manner.

## 2. Materials and Methods

Robotic surgeons from high-volume centers were asked to contribute video content on intraoperative SMA injury. Videos were anonymized, centers deidentified, and dates or times of surgery removed. Surgeons consented for their video material to be used in this study and accompanying video. Patients consented to the video recording of their surgical procedures for education and publication purposes. Three patients underwent left robotic-assisted radical nephrectomy for renal tumors. A transperitoneal approach was performed in two patients, while a retroperitoneal approach was used in one patient.

## 3. Results

The SMA can be injured by either clipping or transection, which could be either partial or complete. The management strategy varies according to the mechanism of injury. Moreover, the common aim is the prompt identification, recognition, and restoration of the anatomical disruption for proper blood supply.

### 3.1. Case #1: Timely Recognition of SMA Misidentification

The left renal hilum is dissected through a retroperitoneal approach. Suspicion was raised when the left renal vein (LRV) was identified posterior to the renal artery. After further dissection, the true left renal artery was identified. Therefore, with timely recognition, injury to the SMA was avoided.

### 3.2. Case #2: SMA Clipping

**Figure 1:**
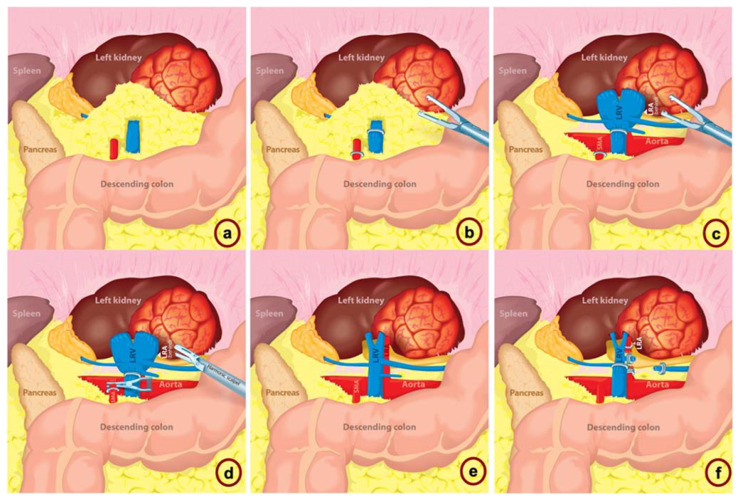
SMA clipping after misidentification as the left renal artery with prompt recognition and management. (**a**): left renal hilum is dissected through a transperineal approach. (**b**): the apparent LRA and LRV are clipped. (**c**): SMA clipped and dilated LRV indicates patent inflow to the kidney. (**d**): a bulldog clamp is placed proximally, and a Harmonic Scalpel is used to remove the clip. (**e**): vascular structures are identified and examined, the true LRA is identified posterior and lateral to LRV, and the SMA is identified anteriorly to the LRV. (**f**): the true LRA is identified and clipped, and the LRV is clipped and collapsed, which indicates no inflow to the kidney. SMA: superior mesenteric artery; LRV: left renal vein; LRA: left renal artery.

Through a transperitoneal approach, the left renal hilum is dissected (Figure 1a). Following further dissection, the apparent left renal artery is identified and clipped. Then, the left renal vein is identified and clipped (Figure 1b). Later, a dilated left renal vein is seen, which raises suspicion for a patent left renal artery, indicating arterial inflow to the kidney (Figure 1c). To manage this adverse event, a bulldog is placed proximally. Using a Harmonic Scalpel, the arterial clip is then removed (Figure 1d). The tip of the harmonic scalpel must be placed carefully to avoid SMA injury. Later, the venous clip is then removed in a similar fashion (Figure 1e). Next, the true renal artery is identified and clipped; following inspection of the SMA, renal vein and artery are examined. The left renal vein has collapsed, which indicates no inflow to the kidney. After confirmation and inspection of the vascular structures, the renal vein is once again clipped (Figure 1f). The error was recognized within 5 min after the SMA clip was placed. The patient was discharged on the postoperative day 2 without further complications.

### 3.3. Case #3: SMA Clipping and Complete Transection

**Figure 2:**
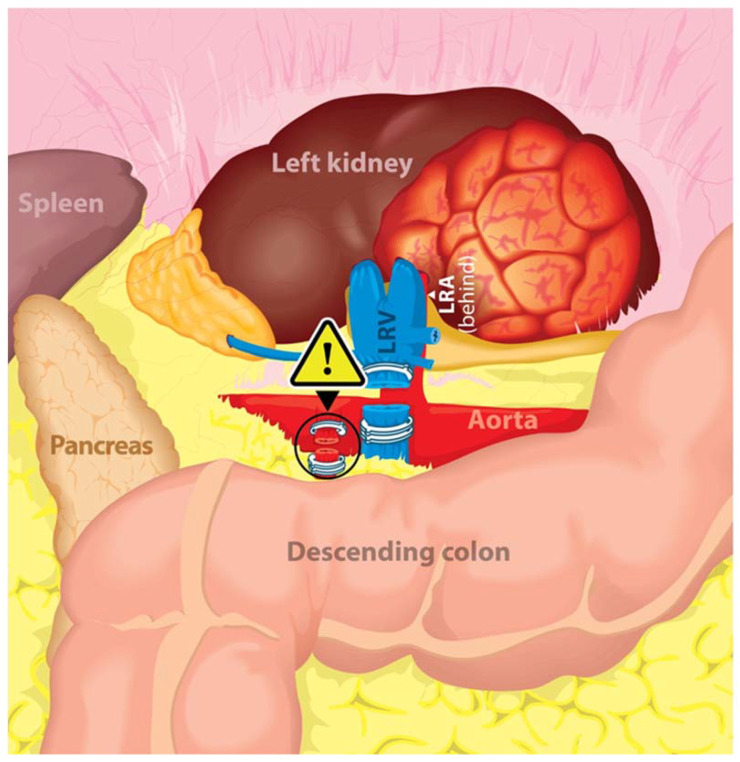
SMA complete transection after misidentification as the left renal artery. SMA: superior mesenteric artery; LRV: left renal vein; LRA: left renal artery.

Through a transperitoneal approach, the left renal hilum was dissected. Significantly distorted anatomy was encountered due to the large left-sided tumor. The SMA, which was located just superior to the left renal vein, was incorrectly identified as the left renal artery, ligated with Hem-o-lok clips, and transected. The left renal vein was then clipped and transected in a similar fashion. Continuing further dissection, an artery arising from the aorta was identified, i.e., the true renal artery. Immediately upon recognition (Figure 2), vascular surgeon assistance was requested immediately. A repair was performed by vascular surgery. A later clinical evaluation of the patient was negative for bowel ischemia, and the patient recovered.

## 4. Systematic Workup Algorithm Management of SMA Injury

### 4.1. SMA Clipping

**Figure 3:**
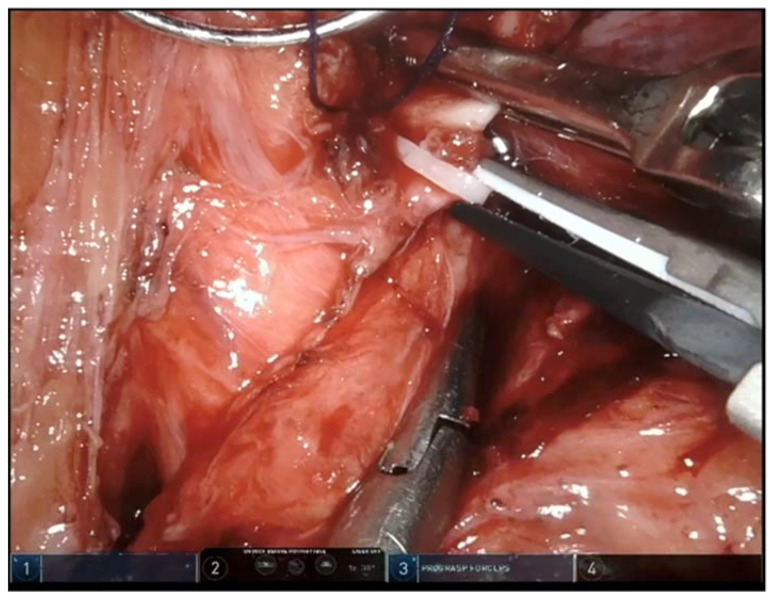
Tip of the harmonic scalpel placed carefully for a precise division of the clip to avoid SMA injury.

**Figure 4:**
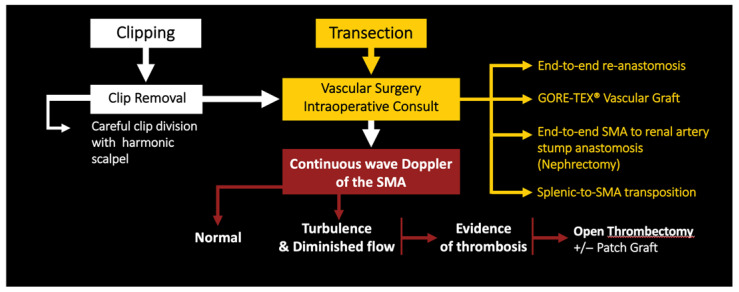
Systematic workup algorithm management of SMA injury.

In cases where the SMA is clipped with Hem-o-lok^®^, the first recommended step is to remove the clip with a harmonic scalpel, which allows for a precise division of the clip, decreasing the risk of damaging the underlying vessel [19]. Therefore, it is advised that the tip of the harmonic scalpel must be placed carefully to avoid SMA injury (Figure 3). Vascular surgery should be promptly informed in case intervention is needed. In addition, continuous wave doppler ultrasound is used to evaluate the integrity of the artery blood flow [20]. In cases where there is a diminished or turbulent blood flow with evidence of thrombosis, an open thrombectomy can be performed [17] (Figure 4).

### 4.2. SMA Transection

If the SMA is transected, vascular surgery should be immediately consulted, and the repair can be performed using several options: (1) end-to-end re-anastomosis, (2) GORE-TEX^®^ Vascular Graft, (3) end-to-end SMA to renal artery stump anastomosis, or (4) splenic-to-SMA transposition [16,17,21,22,23,24]. Surgeons should choose the option with which they are more comfortable and best suits the type of vascular injury (Figure 4).

Of note, it is critical to highlight when to suspect that the SMA can be misidentified as the LRA during a left radical nephrectomy (Table 1). Therefore, it is recommended to follow the artery in its full trajectory to confirm that it enters the kidney (Video S1—Supplementary Materials).

## 5. Discussion

The SMA arises from the abdominal aorta between the celiac trunk and the renal arteries. It provides blood supply to the small and large bowel up to splenic flexure. The SMA provides multiple branches encompassing the inferior pancreaticoduodenal (IPD), middle colic, right colic, jejunal, ileal, and ileocolic artery. It is important to note that communicating arteries exist between the superior and inferior mesenteric arteries (IMA), allowing for multiple pathways of blood supply to the bowel. The major anastomotic network between distal branches of the SMA and IMA is known as the marginal artery of Drummond, while a proximal anastomosis between the middle colic artery (SMA branch) and the left colic artery (IMA branch) is known as the arc of Riolan or meandering artery [25,26].

**Figure 5:**
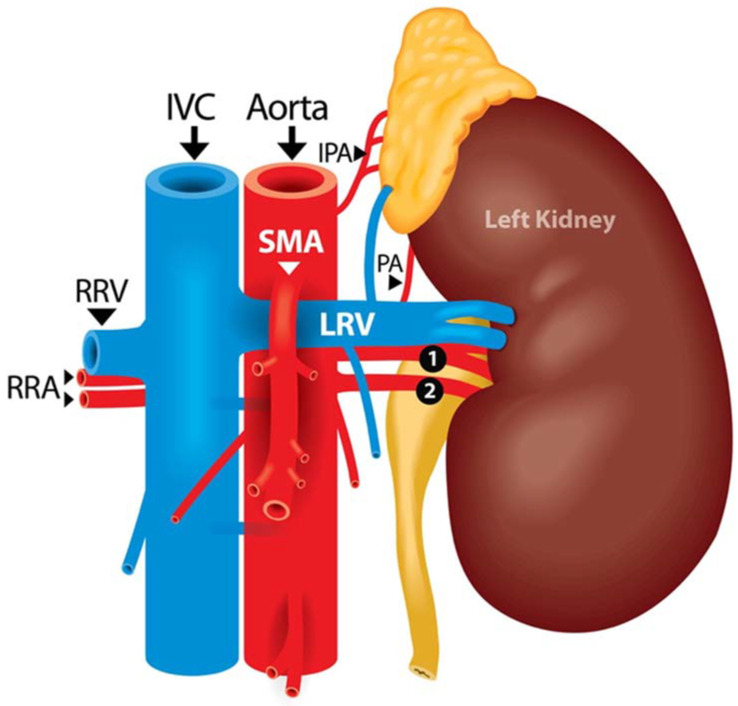
Classic anatomical relationship between the aorta, left renal vein, and SMA. RRA: right renal artery; RRV = right renal vein; IVC: inferior vena cava; SMA: superior mesenteric artery; LRV: left renal vein; IPA: inferior phrenic artery; PA: phrenic artery.

In the classical anatomical relation, the LRV crosses anterior to the abdominal aorta and posterior to the SMA in the crotch of the angle between the SMA and the aorta, the renal artery courses posterior to the LRV, while the SMA drapes over the LRV and can potentially compress the LRV, which is known as the Nutcracker Syndrome (Figure 5). Given the anatomical relationship between the SMA, renal artery, renal vein, and kidney’s location in relation to the abdominal aorta, it is the proximal portion of the SMA that could be more commonly misidentified as the LRA, especially in cases where left renal tumors and/or bulky lymphadenopathy may distort the vascular anatomy increasing the risk of intraoperative vascular injury even for experienced surgeons [11,14,15,16,17]. Therefore, the full identification of the aorta is important in preventing this complication.

A thorough understanding of this surgical anatomy and common variations in renal vascularity is of utmost importance before embarking on kidney surgery [13].

The classical presentation of a single renal artery emerging laterally from the abdominal aorta is seen in only 25% of cases due to great anatomical variability of the renal artery [11], while a retro-aortic left renal vein and circumaortic left renal vein are vascular anomalies that could be encountered in 3% and 3.5% of the cases, respectively [20,27].

Thus, the steep learning curve in minimally invasive surgery and identifying the course of the renal vessels and their relations with SMA and the aorta are essential factors in the appropriate management and prevention of complications during kidney surgery [11,28].

**Figure 6:**
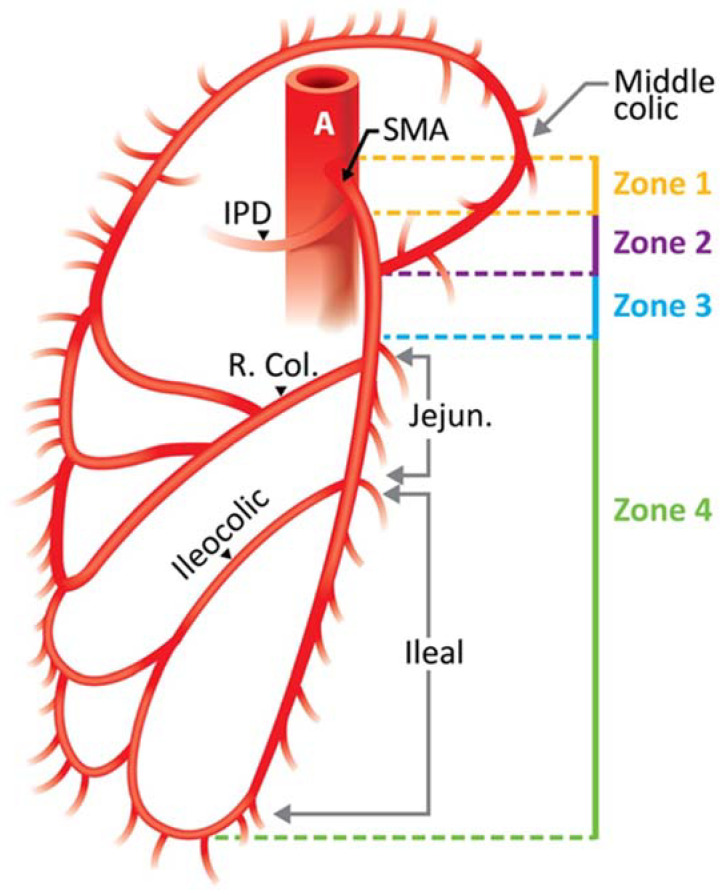
Fullen Anatomic Classification of Superior Mesenteric Artery. A: Aorta; SMA: Superior mesenteric artery; IPD: Inferior pancreaticoduodenal artery; R. Col: Right colic artery.

The location of this type of injury determines the severity of ischemia and is a strong predictor of mortality in models based on traumatic vascular injury [29]; SMA injuries are stratified based on the Fullen classification, which is determined by the anatomic segment of the SMA that is involved [30] (Table 2) (Figure 6). This classification is based on the great number of collateral vessels emerging from the SMA; the level at which the injury occurred can affect the outcome in both bowel ischemia and mortality. Zone I injuries affect the trunk proximal to the first major branch, leading to a greater degree of ischemia, with mortality reported up to 76% [29]. Theoretically, this may be due to the presence of effective collateral circulation in more distal zones. It is important to take into consideration several anatomical variations that have been reported on Drummond’s marginal artery that could jeopardize the collateral circulation at any level or zone of injury predisposing to bowel ischemia, such as an inconsistent right marginal artery, an absent or tortuous marginal artery at the splenic flexure or Griffith’s point, and the absence of the artery at the sigmoid colon in 65%, 7%, and 9%, respectively [26]. All SMA injuries presented in this study are classified as zone I (Table 2) (Video S1—Supplementary Materials).

**Figure 7:**
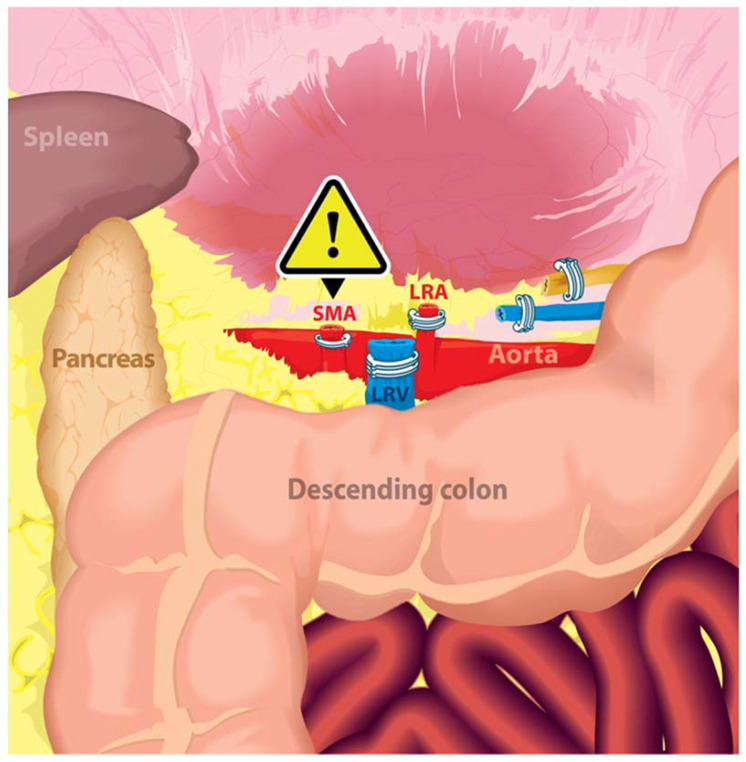
Bowel ischemia following SMA injury during robotic radical nephrectomy. SMA: superior mesenteric artery; LRA: left renal artery; LRV: left renal vein.

Another critical parameter for the successful management of SMA injury is time. This injury inevitably leads to bowel ischemia. Unfortunately, bowel ischemia may not appear until several hours following injury. If not recognized, bowel infarction and necrosis can occur (Figure 7). Severe abdominal pain, metabolic acidosis with elevated anion-gap, and elevated serum lactate may be found due to bowel ischemia [25]. Therefore, it is imperative to perform a delayed inspection for bowel viability and an abdominal CT angiogram in cases where the SMA is injured [31].

Currently, the majority of data reported on this topic is derived from small case series or case reports [11,16,17,24,29], and there are no universally applicable guidelines for assessing, reporting, and managing this daunting intraoperative injury [32,33,34,35,36].

## 6. Conclusions

The proper anatomic recognition of the SMA may prevent its injury. Intraoperative SMA injury should be promptly identified and repaired to avoid its devastating consequences.

## Figures and Tables

**Table 1:** When to suspect SMA.

When to Suspect SMA?
An artery is identified anterior to the renal vein
An artery has an atypical orientation (i.e., transverse)
An artery is medial to the abdominal aorta
More than one large artery encountered, not previously seen on CT
The abdominal aorta has not been fully identified

**Table 2:** Fullen Anatomic Classification of Superior Mesenteric Artery [30].

Zone	Segment of the SMA Involved	Grade	Ischemic Category	Bowel Segment Affected
**I**	Trunk proximal to first major branch	I	Maximal	Jejunum, ileum, right colon
**II**	Trunk between pancreaticoduodenal and middle colic	II	Moderate	Major segment, small bowel and/or right colon
**III**	Trunk distal to middle colic	III	Minimal	Minor segment or segments, small bowel, or right colon
**IV**	Segmental branches, jejunal, ileal, or colic	IV	None	No ischemic bowel

## Data Availability

Not applicable.

## Supplementary Materials

The following supporting information can be downloaded at: https://www.mdpi.com/article/10.3390/jcm12020427/s1. Video S1: SMA Injury during Robotic Radical Nephrectomy: Scenarios and Management Strategies.
